# SNP2SIM: a modular workflow for standardizing molecular simulation and functional analysis of protein variants

**DOI:** 10.1186/s12859-019-2774-9

**Published:** 2019-04-03

**Authors:** Matthew D. McCoy, Vikram Shivakumar, Sridhar Nimmagadda, Mohsin Saleet Jafri, Subha Madhavan

**Affiliations:** 1 Innovation Center for Biomedical Informatics, Georgetown University Medical Center, 2115 Wisconsin Avenue, NW, Suite 110, Washington, D.C. 20007 USA; 2 Radiology and Radiological Science, Johns Hopkins University, 1550 Orleans St, #492, Cancer Research Building II, Baltimore, MD 21287 USA; 3 School of Systems Biology, George Mason University, 4461 Rockfish Creek Lane, MS 2A1, Fairfax, VA 22030 USA

**Keywords:** Molecular dynamics, Ligand docking, Protein structure, Functional prediction

## Abstract

**Background:**

Molecular simulations are used to provide insight into protein structure and dynamics, and have the potential to provide important context when predicting the impact of sequence variation on protein function. In addition to understanding molecular mechanisms and interactions on the atomic scale, translational applications of those approaches include drug screening, development of novel molecular therapies, and targeted treatment planning. Supporting the continued development of these applications, we have developed the SNP2SIM workflow that generates reproducible molecular dynamics and molecular docking simulations for downstream functional variant analysis. The Python workflow utilizes molecular dynamics software (NAMD (Phillips et al., J Comput Chem 26(16):1781-802, 2005), VMD (Humphrey et al., J Mol Graph 14(1):33-8, 27-8, 1996)) to generate variant specific scaffolds for simulated small molecule docking (AutoDock Vina (Trott and Olson, J Comput Chem 31(2):455-61, 2010)).

**Results:**

SNP2SIM is composed of three independent modules that can be used sequentially to generate the variant scaffolds of missense protein variants from the wildtype protein structure. The workflow first generates the mutant structure and configuration files required to execute molecular dynamics simulations of solvated protein variant structures. The resulting trajectories are clustered based on the structural diversity of residues involved in ligand binding to produce one or more variant scaffolds of the protein structure. Finally, these unique structural conformations are bound to small molecule ligand libraries to predict variant induced changes to drug binding relative to the wildtype protein structure.

**Conclusions:**

SNP2SIM provides a platform to apply molecular simulation based functional analysis of sequence variation in the protein targets of small molecule therapies. In addition to simplifying the simulation of variant specific drug interactions, the workflow enables large scale computational mutagenesis by controlling the parameterization of molecular simulations across multiple users or distributed computing infrastructures. This enables the parallelization of the computationally intensive molecular simulations to be aggregated for downstream functional analysis, and facilitates comparing various simulation options, such as the specific residues used to define structural variant clusters. The Python scripts that implement the SNP2SIM workflow are available (SNP2SIM Repository. https://github.com/mccoymd/SNP2SIM, Accessed 2019 February ), and individual SNP2SIM modules are available as apps on the Seven Bridges Cancer Genomics Cloud (Lau et al., Cancer Res 77(21):e3-e6, 2017; Cancer Genomics Cloud [www.cancergenomicscloud.org; Accessed 2018 November]).

## Background

Molecular simulation is a powerful tool used by computational biologists to analyze the relationship between protein structure and its functional properties. Ranging from high throughput drug screening to focused characterization of protein conformational dynamics, the creative analysis has several translational applications. Large libraries of drug candidates can be evaluated to produce novel targeted therapeutics, and insight into specific molecular interactions between effective drugs and their protein targets aids the design novel molecules [1, 2]. An advantage of the computational simulations is the ability to probe how variation in the protein sequence alters those molecular interactions, and can be extended to the development of therapies targeted at specific sequence variants [3–6]. In addition to drug discovery and design, the insight can be further extended to inform treatment planning when selecting an optimal targeted therapeutic strategy [7].

Due to an inherent tradeoff between resolution and computational requirements, molecular simulations can be divided between approaches which only simulate a fraction of the overall molecule and those which explicitly consider all atomic interactions occurring within a solvated system. Coarse grained methods which do not explicitly consider the internal interactions occurring within the protein backbone are used to address the enormous search space that must be sampled when predicting how two molecules interact [8]. For example, predicting how well a small molecule ligand will bind to a target protein depends on the sum total of all the individual atomic interactions. Depending on the chemical nature of the ligand, the conformational diversity can be quite large due to rotation around individual bonds and limited steric constraints of a single ligand molecule. Furthermore, the protein surface represents a large area of potential interactions and exponentially increases the degrees of freedom which must be explored when identifying an optimally bound structure. In order to simplify the search for optimized protein:ligand conformations and to simulate high throughput binding of large libraries of low molecular weight ligands, coarse grained docking methods will typically only model the flexibility of the ligand and a small number of interacting protein residues within a defined area of a rigid protein structure [8].

While the liberties taken by these types of simulations allow for a greater throughput, they fail to account for internal protein dynamics which may play a significant role in the interacting complex. All-atom molecular dynamics (MD) simulations explicitly account for atomic interactions occurring within a molecular system and provide a way to understand the overall conformational flexibility and structural dynamics [9]. However, even systems consisting of a small, solvated protein contain tens to hundreds of thousands of atoms and each simulation step requires a summation of all the forces acting on each. Even on high performance computational infrastructures, simulation runs can easily last weeks to generate usable results. The increased computing cost is offset by its unique insight and characterization of functionally relevant protein dynamics.

Both approaches find utility in specific applications, and their individual strengths are leveraged to understand the impact on protein sequence variation on small molecule binding. Upon mutation of a residue, the change in the amino acid side chain has the potential to alter the functional interactions with a small molecule. If the change occurs within the defined search space of a coarse grained binding simulation, the new interactions can be simulated directly. Typically, the structures used for binding simulations are derived from x-ray crystallography, but simply swapping out amino acid side chains in the intersecting residues may not fully account for the structural differences of the protein variant. Since the protein backbone is treated as a rigid scaffold, the predicted binding characteristics do not account for those subtle changes in the backbone geometry and could have a large influence on the results. Furthermore, these methods have nothing to offer if the variation occurs outside of the defined search space, especially those amino acids which are buried within the folded protein structure. MD simulations can address this limitation by comprehensively sampling the conformational landscape of a protein variant to generate characteristic scaffolds for downstream small molecule docking.

**Fig. 1:**
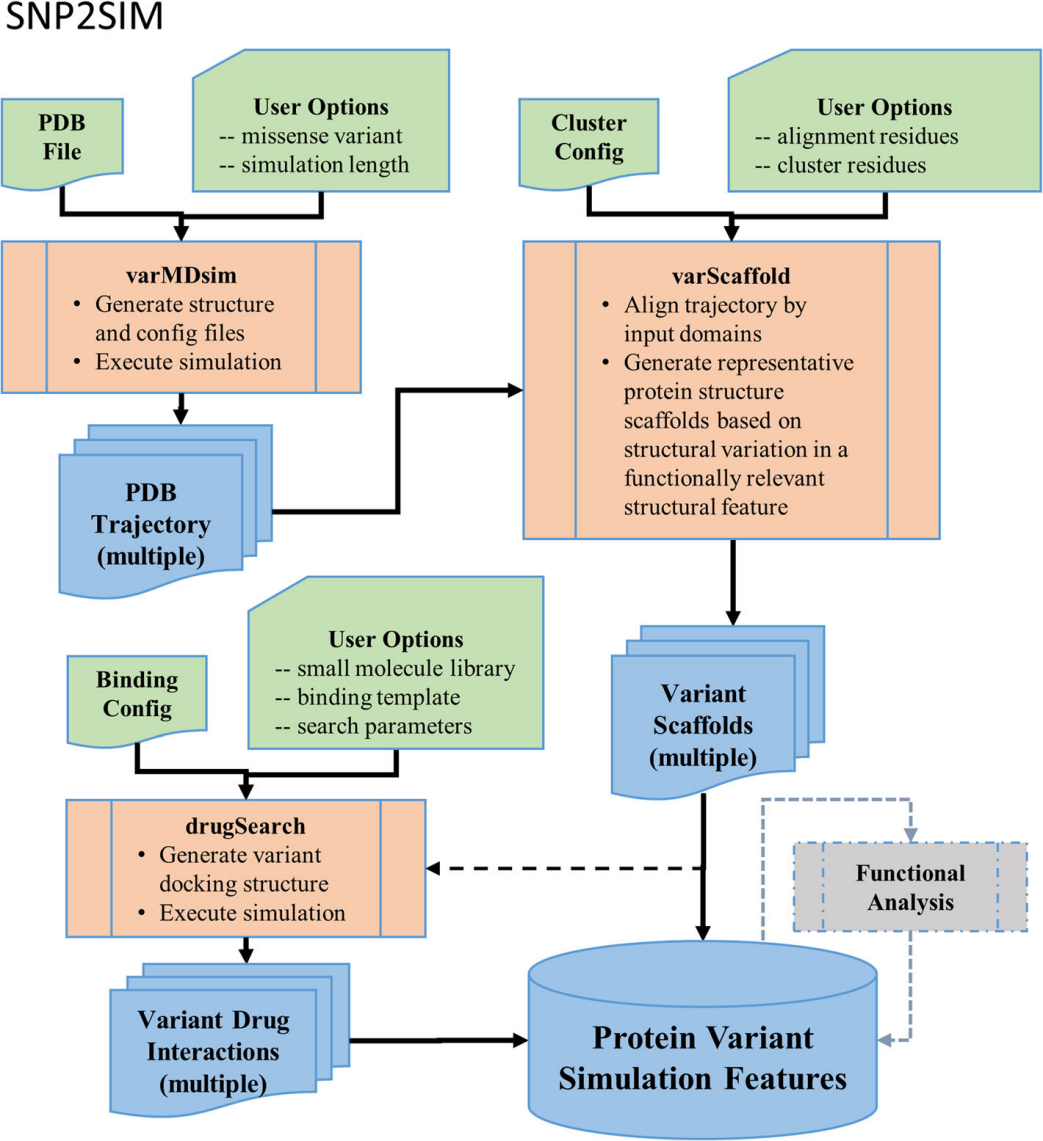
The SNP2SIM workflow contains 3 functional modules (shown in orange) that execute all atom molecular dynamics of protein structure variants using NAMD and VMD (varMDsim), clusters the resulting trajectories into a set of structures that represent the conformational dynamics of the binding interface (varScaffold), and predicts the binding interactions of low molecular weight ligands using AutoDock Vina (drugSearch). The input for each module (green) control their configuration, providing a way to standardize simulation parameters across parallel computational infrastructures. The resulting structural datasets (blue) can be used to analyze protein:ligand interactions and enables large scale investigations into the functional consequences of protein sequence variation

Since a protein variant can alter the functional interaction with therapeutic molecules, predicting how small molecules will bind to protein variants has a significant application in personalized medicine. Not only can simulation results be used in the development of targeted therapies, it could also be informative in the selection of second line of therapy once drug resistance has emerged. As the application of molecular profiling and sequence analysis continues to gain a foothold in clinical decision making, a well-defined, user friendly simulation workflow and methodology will continue to be an important tool for translational computational biology. To that end, we present SNP2SIM (Fig. 1), a scalable workflow for simulating the impact of protein sequence variation on binding to small molecule ligands.

## Implementation

At its core, SNP2SIM is a modular set of simulation and analysis tools wrapped in a command line Python script. There are many molecular dynamics simulations packages available, and the backend of the SNP2SIM workflow is designed to easily incorporate additional simulation packages in the future to customize the workflow and better accommodate user preferences. This initial implementation is built around Nanoscale Molecular Dynamics (NAMD) [10] and Visual Molecular Dynamics (VMD) [11] due to their scalability, interoperability, and implementation across a wide range of high performance computing infrastructures and operating systems. VMD is also used to process the results of the NAMD simulations and cluster the resulting trajectories according to structural variation in the protein:ligand binding interface. A representative conformation from each cluster is chosen to create a set of variant specific protein structures that reflect the subtle changes to its conformational diversity. AutoDock Vina [12] is used to perform the small molecule docking, and was selected due to its widespread use, ease of implementation within the workflow, and computational performance.

Starting with only a PDB formatted file of the protein structure, three independently run functional modules perform the molecular dynamics simulation of a protein variant, cluster of the resulting trajectories based on conformational variation in user defined binding residues, and dock small molecule ligands into each variant specific structural scaffolds. The workflow is designed to be used as a tool to aid large scale computational mutagenesis studies, enabling uniform application of simulation and analysis parameters. SNP2SIM minimizes the simulation options exposed to the user to control the generation of tool specific preprocessing and analysis scripts, define the parametrization options used in the configuration files, and output simulation results into a predefined file structure. The standardized file structure and naming conventions provide the option to implement the modules across independent computational systems and easily aggregate the results for downstream analysis.

The command line implementation of SNP2SIM is available for download from a GitHub repository [13], and the varMDsim, varScaffold, and drugSearch modules are also available as apps on the Seven Bridges Cancer Genomics Cloud [14, 15]. Due to the nature of MD simulations, the computational requirements of the workflow are dependent on the overall size of the protein structure and can grow to become quite significant, even on high performance infrastructures.

### varMDsim

With the minimal input of a PDB formatted protein structure file and simulation time in nanoseconds, the varMDsim module will generate a solvated, ionized water box around a mutated protein structure, create the configuration files for the all-atom, explicit solvent simulation with periodic boundary conditions, and compile the results for downstream analysis. Utilizing the VMD Mutator, Solvate, and Autoionize plugins, the workflow will automatically mutate the input structure prior to solvation. The CHARMM36 force field [16] is used to parameterize the protein structure, and water molecules use the TIP3P water model. The simulation configuration files are hardcoded into the workflow, standardizing the resulting simulation for reuse and promoting the reproducibility of the computational simulations.

The run length of simulations is highly dependent on the nature of the protein under study, and can become significant for highly dynamic or large structures. However, since the aim is to capture subtle, variant induced changes to the conformational dynamics of the ligand binding interface, the structural diversity should be sufficiently sampled after hundreds of nanoseconds. Since SNP2SIM is configured to run the version of NAMD (including those utilize GPUs) installed on the user system, the varMDsim module can first be applied to benchmark performance.

### varScaffold

The simulation trajectories are analyzed using the varScaffold module to produce characteristic structures of protein variants. More than merely clustering the collection of protein structures from the MD simulations, varScaffold first aligns the entire set to a common reference frame (typically over the entire protein structure) before measuring the root mean square deviation (RMSD) in the backbone of a subset of amino acids involved in ligand binding. Using the VMD “measure cluster” command, where a user supplied RMSD threshold is used to identify the 5 most populated configurations of the binding residue geometry. If one of the clusters is assigned a significant portion of the overall population of simulated results, a representative structure is chosen as a variant scaffold for downstream ligand binding.

The varScaffold module will accept multiple PDB or DCD formatted trajectory files generated through parallel execution of the varMDsim module. Since the clusters are determined using a relatively small number of residues, the number of populated clusters is very sensitive to the RMSD threshold. The workflow enables the iterative application of clustering parameters, allowing the user to specify which binding residues are used to define the binding interface geometry and determine the optimal RMSD cutoff before applying the module to the entire variant population.

### drugSearch

The drugSearch module uses AutoDock Vina [12] to bind a library of low molecular weight molecules into the variant scaffolds. Unlike the previous modules which are largely automated, the configuration of the drugSearch module requires the user to define the ligand binding site on a reference structure. This requires the user to supply a PDB formatted protein structure (typically the structure used to initiate the varMDsim module), and an associated parameter file that defines the coordinates and dimensions of the search space. Additionally, the user can specify a set of residues within that search space model with flexible sidechains. These search parameters can be determined using the AutoDockTools software package, which accompanies the AutoDock Vina distribution.

The drugSearch module streamlines the process of ligand screening by aligning the individual variant scaffolds to the reference coordinates, generates the AutoDock Vina structural input and associated configuration files, and sequentially predicts the binding interactions and energies for individual ligands in the specified drug library. Several large libraries of ligands from The National Cancer Institute Developmental Therapeutics Program (Diversity Set 5, Mechanistic Set 3, and Natural Products Set 4) are included in the SNP2SIM repository, and additional libraries can be easily incorporated. The drugSearch module outputs the coordinates and binding energies for the top 9 high affinity poses for each small molecule.

## Results

The immunomodulatory protein programmed death ligand 1 (PD-L1) was used to demonstrate a typical application of the SNP2SIM workflow to drug development in immunotherapy. In some cancers, overexpression of PD-L1 leads to inactivation of the immune cells that attack the tumor, leading to the development of small molecule inhibitors that selectively inhibit PD-L1 interactions [17–20]. To understand how these molecules may differentially bind to variants of PD-L1, known mutations in the binding domain were processed through the SNP2SIM workflow. The initial starting structure used the Ig-like V-type domain from PDB: 4Z18, and 5, 100 ns simulations were generated for a set of protein variant found in common experimental cell lines, as well as those most commonly occurring across all cancer types (L53P, V68 L, L94 M, G95R, A97V, M115 T) [21]. Variant trajectories were aligned using the entire domain backbone and clusters were defined using a 0.7 Angstrom RMSD cluster threshold for the backbone atoms in residues interacting with low molecular weight inhibitors in PDB crystal structures [17–20] (Residues 19, 20 54, 56, 66, 68, 115, 116, 117, 121, 122, 123, 124, 125). These same interacting residues were also modeled with flexible side chain torsions. The SNP2SIM workflow was run using the Seven Bridges Cancer Genomics Cloud infrastructure [14, 15], and the files needed to run this example are provided in the SNP2SIM code repository [13].

**Fig. 2:**
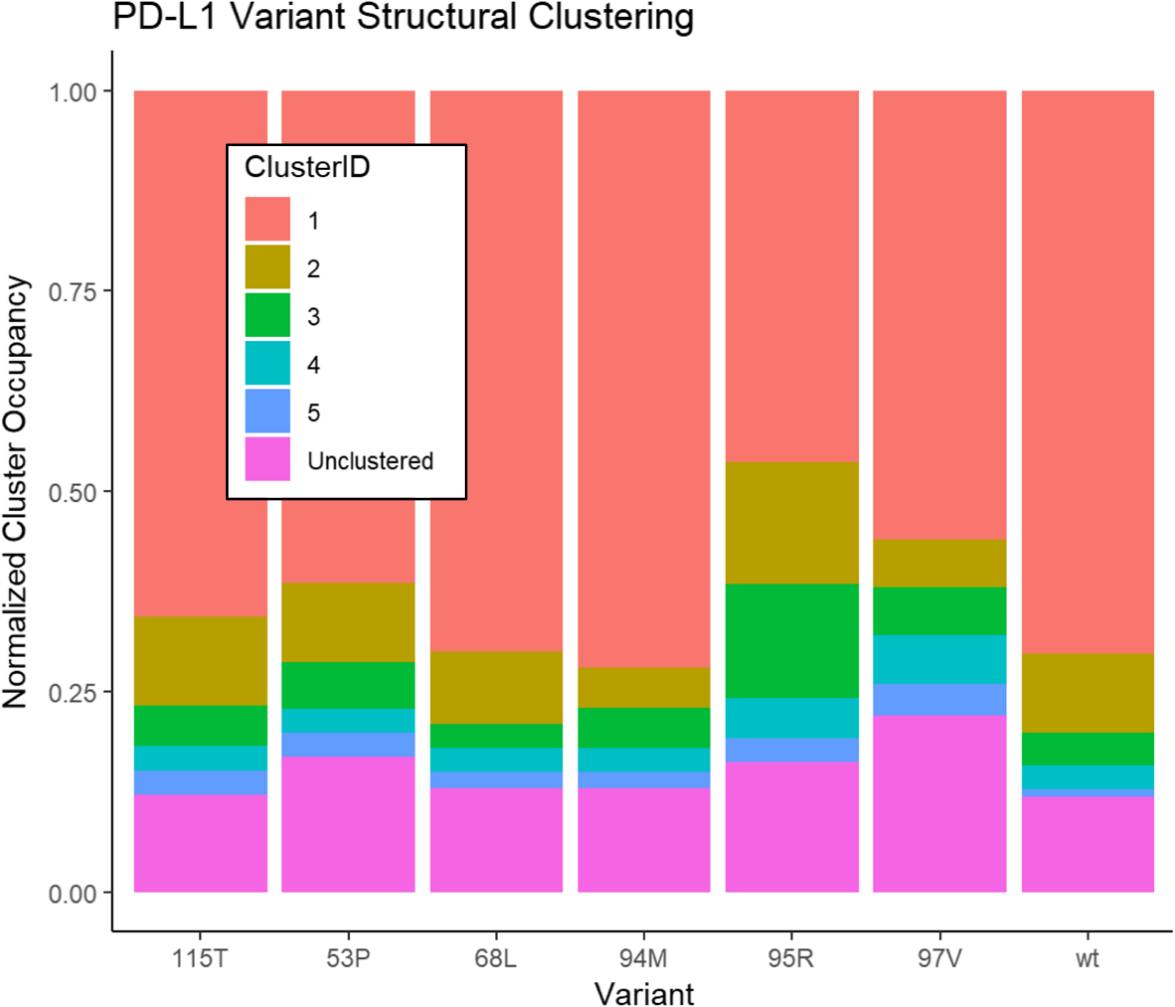
The breakdown of the results from the varScaffold module of the SNP2SIM workflow show the variation induced changes to the organization of the PD-L1 binding residues in the simulated structures. The clusters are ranked by the total number of MD conformations that fall within the user supplied RMSD threshold, and the remaining structures that are not assigned to the top 5 clusters are given the “Unclustered” designation. A representative structure from each cluster that contains at least 10% of the total structures derived from the simulated trajectories are used to create a representative scaffold for drug binding

As demonstrated through the PD-L1 case study, the SNP2SIM workflow enables the efficient parallelization of the computationally intensive molecular dynamics simulations and streamlines the generation of variant specific protein structure scaffolds for ligand binding. The MD simulations were parallelized across 5 independent runs, and integrated using the varScaffold module. The resulting structural clusters (Fig. 2) show that certain variants induce more conformational flexibility than others. The wildtype PD-L1 structure had two clusters populated by at least 10% of the simulated trajectory structures. Depending on the variant, the number of structural clusters that lead to binding scaffolds decreased to one (94 M, and 97 V), increased to three (95R), or stayed the same (53P, 68 L, and 115 T), illustrating the differential impact of sequence variation on the overall conformational flexibility.

**Fig. 3:**
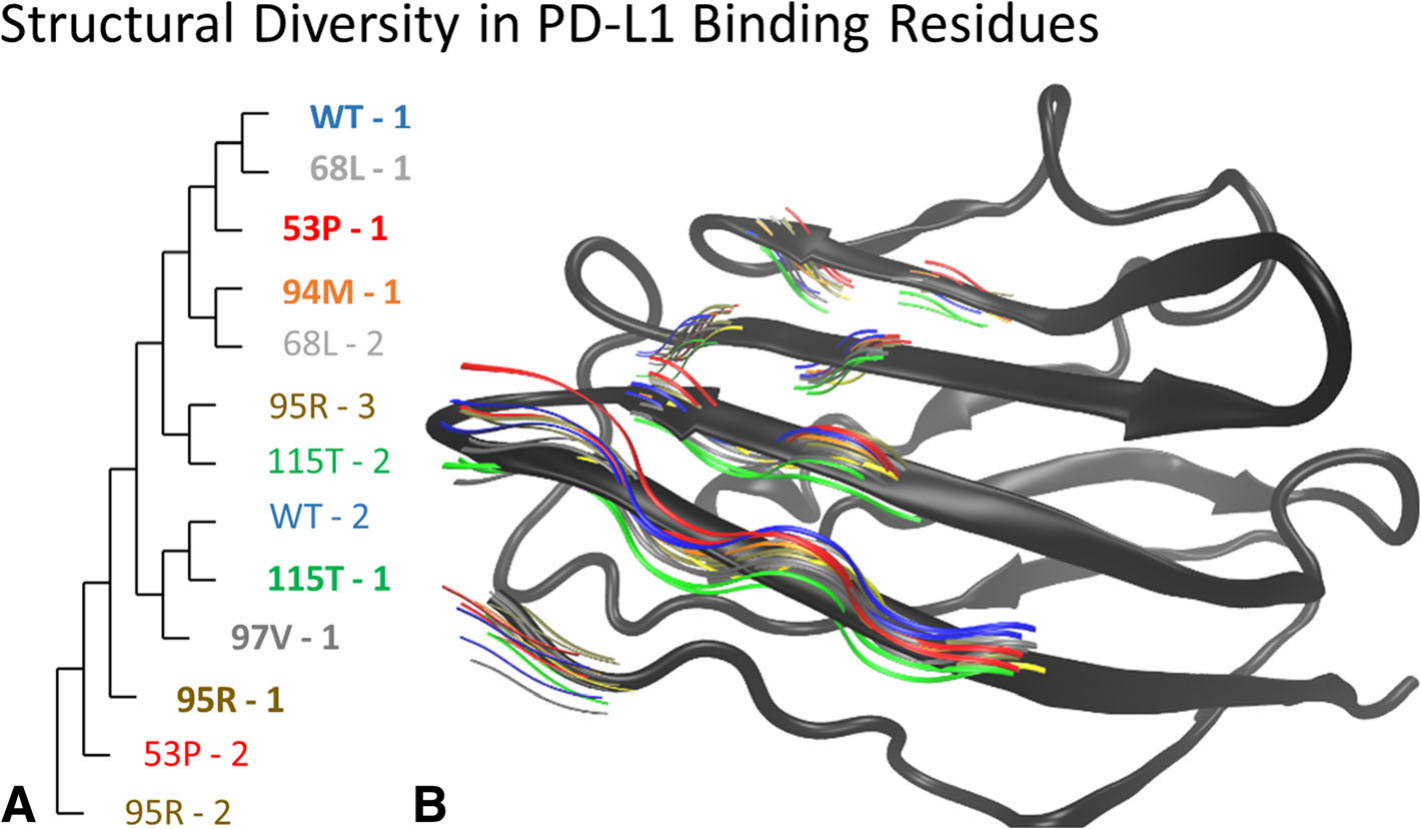
**a**. The multiple structure alignment of scaffolds generated for PD-L1 variants shows the divergent impact of the amino acid substitution on the protein structure. The variants are annotated with the rank (1, 2, or 3) that corresponds to the relative proportion of the MD structures that occupy that structure. **b** When the representative scaffolds are aligned to the initial crystal structure (grey), the conformational changes of the PD-L1 binding residues show the divergence of the variant structural scaffolds from those derived from the wildtype simulations (blue)

The representative structures can be analyzed to gain insight into how they variant structures relate to each other. When aligned over the protein backbone from the initiating experimental structure, the range of variant induced conformational flexibility can be seen in the relative positions of the PD-L1 ligand binding residues, and structural clustering using multiple protein structure alignment [22] reveal how the most populated variant structures (95R-1, 97 V-1, and 115 T-1) are structurally divergent from the most populated wildtype conformation (Fig. 3). The differences in flexibility translate to changes in the predicted binding affinity to an interacting ligand, and can be used to predict if a given drug will be more or less likely to bind to a protein variant.

**Fig. 4:**
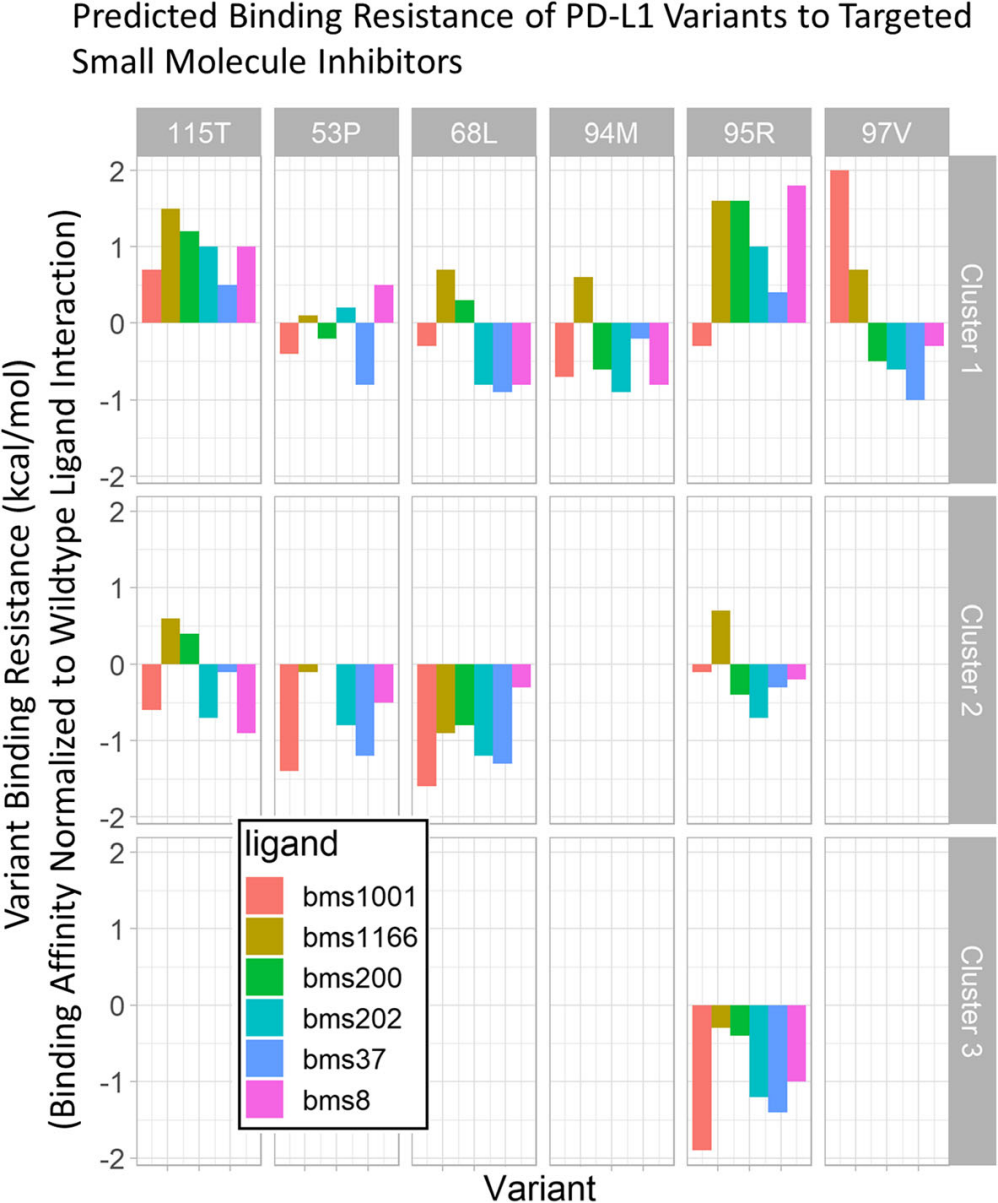
The SNP2SIM drugBinding results for trajectory-derived PD-L1 variant scaffolds can be used to compare the binding affinity of the wildtype structures to that predicted for the structural variants. By normalizing to the wildtype prediction, the relative resistance of variants to a selection of PD-L1 inhibitors can be quantified. Since lower energies correspond to stronger molecular interactions, the drug resistant variant will have a higher binding affinity than the wildtype, and a positive value on the plot

An initial indication of the potential of a variant to disrupt binding can be determined by comparing the predicted binding affinity of the variant structure to the affinity the wildtype [7]. The results from the drugSearch module were used to generate the plots of predicted variant drug resistance in Fig. 4. The results for only the most populated wildtype structure are shown, but the comparison of the variant scaffolds to the other wildtype scaffold showed a similar pattern. The results show that the most populated variant clusters (Cluster 1) can be more disruptive to binding than others, for example the 115 T and 95R variants both seem to disrupt binding to all the ligands. Additionally, different structural clusters for the same mutation can show divergent behavior, the most prominent example being the difference between Cluster 1 and Cluster 3 for the 97 V variant.

## Discussion

The growing prevalence of genomic testing is revealing an enormous amount of rare variants with unknown functional significance [23], underscoring the need for predictive computational analysis to determine their biological impact. This is especially true for variants which occur in proteins where the effectiveness of targeted therapeutic strategies may be disrupted. For example, missense mutations that emerge in response to evolutionary pressures in a growing tumor to disrupt binding of targeted inhibitor molecules [24]. SNP2SIM enables the profiling of multiple approved inhibitors to inform the selection or design of an optimal therapy that maintains a positive clinical response [7].

By simulating the variant specific contributions to the overall protein conformational dynamics and ligand binding, the unique impact of a variant can be quantified even when the mutated residues do not occur at the interaction interface. This offers an advantage over using the crystal structure as the basis for small molecule docking simulations, instead providing a set of structures that is specific to the impact of the given variant. This is significant, as MD can capture conformational states not represented in crystal structures [25]. Even for the wildtype structure, two populated conformations were identified which show slightly modified geometries of the protein backbone found in the crystal structure.

## Conclusions

The SNP2SIM workflow represents a higher resolution approach to in silico ligand binding. Instead of using a single structure derived from crystallography experiments, a set of variant specific scaffolds are used to predict the binging affinity to small molecule ligands. The additional information on protein dynamics will ultimately produce more robust analysis and improve predictive models used for downstream drug development, design, and utilization. While the current iteration of SNP2SIM only manages the execution of the simulation workflow, predicative models can be built that integrate the data on the population (Fig. 2), structural divergence (Fig. 3), and binding interactions (Fig. 4).

The utility of a predictive, simulation based model, and the insight it can provide to understanding the functional changes of protein sequence variants, is rate-limited by computational costs and scale of potential variation. PD-L1 was chosen because it presented an optimal development case, where the size and structural stability helped to minimize the computational time required by the MD simulations. When simulated larger domains, such as folds that result in the ATP binding pocket in protein kinases, the computational requirements to generate relevant simulation timescales can grow to become prohibitive. These barriers are being overcome through access to cheap cloud computing and the development of reproducible workflows that can integrate standardized results from multiple research groups. And while a lot has been done to lower the barrier for novice users to access these tools through widely available infrastructure such as the NCI cloud pilots, creating an easy-to-use simulation and analysis workflow opens the doors to many researchers who would otherwise not have access. SNP2SIM ensures a uniform generation of input files, application of simulation parameters, and quantification of the results, and enables the parallel implementation of molecular simulations across hardware infrastructure.

## Availability and requirements

Project name: SNP2SIM.

Project home page: https://github.com/mccoymd/SNP2SIM

Operating system: Linux.

Programming language: Python.

Other requirements: Nanoscale Molecular Dynamics (NAMD), Visual Molecular Dynamics (VMD), AutoDock Vina, AutoDock Tools.

License: FreeBSD.

Any restrictions to use by non-academics: Yes, subject to license and usage agreements for simulation software packages.

## Abbreviations

MD: Molecular Dynamics
NAMD: Nanoscale Molecular Dynamics
PD-L1: Programmed death ligand 1
RMSD: Root mean square deviation
VMD: Visual Molecular Dynamics
